# 

**Published:** 1922-01

**Authors:** 


					﻿SUBJECT INDEX TO VOLUME LXXVI
January, from page 1 to 50.	FORMAL PAPERS
February, from page 51 to 98.	Art and Ethics as Applied to
March, from page 101 to 147.	' rosthetics. By E. P. Lowery,
April, from page 151 to 196.	A	fOr Gold Foil. Bv G. B.
May, from page 201 to 246.	Baird. D.D.S., 484.
June, from page 251 to 300.	A Reliable Hemostat. By Alfred
Julv, from page 301 to 350.	F. Hess, M.D., 375.
*	.	,	. ono	A Three, lour or Five xear
August, from page 3ol to 398.	Course.	p R Bremnerj
September, from page 401 to 448. D.D.S. 15.
October, from page 451 to 498. Banquet to John P. Buckley, 68.
November, from page 501 to 524. Bridge M orU ixed and Remov-
,,	,	,	,	able. By E. P. R. Ryan, D.D.S.,
December, from page 52a to —.	jgg J	J ’
Building the Structure of Our
_________ Lives. By George Wood Clapp,
D.D.S., 281.
Capping the Pulp. By R. Otto-
EVENT AND COMMENT	lengui, D.D.S., 369.
.	.. .	,	,,,	Casting Processes as Viewed by
A Chi dren s Health Drive, 501. the Metallurgist. By M. M.
Annual National Dental Associa- Wilkinson, E M.M.S., 227.
tion, 451.	Consideration of Abscesses the
Annual Ohio Dental Society, 52a.	Mouths of Children. By F. W.
Balance in Dental Education, 2a2.	Hnlliq+pr DMD 338
Dental Hygiene, 555.	Consideration 'of Some Matters
Dentistry and the L nfortunate Pertaining to Occlusion and the
Poor, oo3.	Occlusal Performance. By James
Is Dental Radiography Malprac- Kendall Burgers, D.D.S., 572.
tice? 201.	Dental Infection Secondary	to
Memorial Monument to Chopin A. Maxillary Sinusees. By John
m8’	A. Glassburg, M.D., 220.
Ohio State Annual	Meeting, 551.	Dental Prophylaxis. By T.	P.
Our Filling Materials, 51.	Hyatt, D.D.S., 284.
Partial Dentures, 302.	Diseases of the Teeth and Mouth
Pioiessional or Commercial Stand- as Causes of Organic Diseases,
ards, 401-	Metropolitan Insurance Bulletin,
Recruation Requirements, 351.	1922 225
The treatment and lilling of Essential Factors in Denture Con-
Deciduous Teeth. By M. Evange- struction. By Norman S. Essig,
line Jordan, D.D.S., 555.	D.D.S., 2.
The Next Move in Dental Educa- Failure of Denture Methods and
tion, 101.	a Simple Remedy. By Stewart
Value of Diagnosis as a Factor A. Spence, D.D.S., 131.
in Dental Health, 151.	Healthy Living and Recreation.
What Will the New Year Do for By George Wood Clapp, D.D.S.,
Dentistry? 1.	354.
Is Apicoectomy Ever Successful? The Dignity and Importance of
If so Under What Conditions? Dentistry. By Edwin C. Kirk,
By Drs. Eby and Jaffer, 324.	D.D.S., 177.
Is Pyorrhoea a Local or a Con- The Full Lower Denture and the
stitutional Disease? By Russell Substitute Offered. By Elbert
W. Bunting, D.D.S., 302.	B. Owen, D.D.S., 211.
Medical Training of Dental Stu- The Incidence of' Cancer. Journal
dents. By Thomas B. Hartzell, A. M. A., 380.
M.D., D.D.S., 265.	The Metallurgy of Platinized Gold
Occupational Dermatitis. By A. S. Cast Clasps. By M. Elisberger,
Walker, M.D., 61.	367.
Our Task. By C. V. Littler, The Nature and Manipulation of
D.D.S., 21.	Amalgams and Its Standardized
Oral Prophylaxis versus Sarcomas. Technique. By R. K. Brown,
By M. Rattner, D.D.S., 253.	D.D.S., 153.
Periodontoclassia. By John O. The Psychological Factor in Secur-
McCall, D.D.S., 127.'	ing Central Occlusion. By Day-
Preventive	Dentistry; Its	Public	ton Dunbar	Campbell,	D.D.S.,
Aspect.	By Edwin N.	Kent,	U3.
D.M.D., 508.	The Result of Five Years’ Prophy-
Preventive	Dentistry.	By	A. S.	Iaxis in Metropolitan Life Co.’s
Hopkins,	D.D.S , 82.	Office- By T-	p- Hyatt,	D.D.S.,
Proper Treatment of Enamel. By ^6.
J.	P. Carmichael, D.D.S., 218. The Hole of the Tooth Brush in
Prophylactic Value of Nitrous	^os4’
Oxid-Oxygen in Extracting to D.D.S., 184.
Avoid Systemic Reactions. By Treatment of Edentulous Jaws for
II. H. Harms, D.D.S., 6.	Efficient Service.	By	G. W.
Radiodontia, Its Uses and	Abuses.	Dittmar, D.D.S.,	113.
By Herbert G. Frankel, D.D.S., The Swinging Back of the Pendu-
272	lum. Editorial	in	Dental
Restoring Teeth in Porcelain Cosmos, Dec. 1921, 34.
Without Shoulder Preparation. Two Years’ Experience with a
By Albert L. Lero, D.D.S., 204.	Dental Program. By John C.
Some Chemical Problems as Ap-	Gebhart, 331.
plied to Dentistry. By Albert Under What Conditions May Epi-
K.	Epsteim, 418.	oectomy be Successful? By C. J.
Some Important Factors which Lyons, D.D.S., 260.
Influence Occlusion. By G. S'. Use of Radiography in Dentistry.
Monson, D.D.S., 317.	'	By J- H- Taylor, D.D.S., 505.
Surgical Preparation for Pros- Valedictory Address. Bv Stanley
thetic Restorations. By Drs.	L(’n^’ ? DK’ 2I6’ + n
Giffen, Rogers and Kemper, 103.	V hat Denture service posts. By
The Barnes Method of Brushing ^1°^	°°d C app’ ‘ ’S‘’ 54’
^By Henry BarneS’ What" Should be Our Ideal of
B.1J.O., 17.	Prosthetics? By W. F. Chappelle,
The Dentist and the Laboratory.	D.D.S., 360.
By C. H. Reese, D.D.S., 58.	When to Extract and When to
The Dentist’s Responsibility Dur-	Conserve Diseased Teeth. By
ing Gestation. By Arthur H.	Thomas	B. Hartzell, M.D.,
Nobbs, D.D.S., 120.	D.D.S., 77.
INDENTS	Injudicious Extraction of Teeth,
416	'	’	Intelligence and Decision m Diag-
Acid Mouth Washes. Dental Facts, nosis- BX c'■ 01se^ 439.
40	Importance of Correct Radiograms.
A Fiuid Flox that Does Not Pit. T Bv ’ A‘ fLlTirie’ 444/. , ,r .
Oral Health, 142.	Importance of Lower Third Molars.
Alcohol as a Surgical Disinfect By Wm. M. Stanbrough, 43.
ant 588 urSlcal Lisiniect Jap observations of Dental Edu-
Annealing Clasp Gold. Bv F. T.	•	t> a a
Drescli, 293.	*	Matr?x. Retai"7Q B? A’ S‘
A New Idea in Communitv Dental AT-	,. , c .	.
o t i	o* i, 4.	Michigan State Medical School.
Sepee. By John D. Robertson, Jnu;„a| x M A> ]J3
Business Building. F. C. E„ in	Extraction and Coropli- .
Digest _____1_	cations. By Julio Endelman,
Candv. Oral Hvgiene, 341.	o
C\?PALrE4lr1 PUlPS' 87 "• R. H.Os£ngO,13PiyS,ClanS-
Care of’Hie Teeth. Public Health Nomenclature Report. 481.
Reports 341	National Dental Association, 145.
Concerning the’ Etiologv of Pvor- Observations on Radiography, 342.
rhoea. By Julio Endelman, 139. Occupational Dermatitis. Uy H.
Conserving Public Health, 583.	j	* V, ...	. ,T
Cost and Receipts in Practice. Bv Oral Hyg>™ C™™ttee of New
G W Clann 133	Vork- B.v T- P- Hyatt> 287.
Dental Ethics 46	Oral Prophylaxis Chart By Best
Dental Fatigue. By W. H. De- a"d Maldron, Journal N. D A.
Ford 88	Our Knowledge of Vitamins, 194.
Determination of Injection. By Practical Hints for Oxygen Gas
A. B. French, 84.	Extractions. By George C. Cox,
Epilepsy from Lower Third Molar n ‘	• i	t o ,
Extraction. By W. Gauley, 443. Preventing Shrinkage when Solder-
Endowment for Hopkins Hvgiene T> "??• v	V !vie’ ^2‘ t>
School 190	Radium for Dental Abscesses. By
X-Rav, Perhaps. Bv C. K. Field, n J:. A’ Mar«baB, 294.
350	J	Radiograph Discussion. By Dr.
Extracting and Pregnancy. By n RoR,a’ 34f’-	.	.
W. N. Rowlev 2927	Pe" vulcanite Dentures Injurious,
Extracting Infected Teeth, 585.	.
Facts About Tuberculosis, 522. Removing Trays from Compound,
Figures Never Lie, Figurers Do. P3’
Journal A. M. A., 236.	Relief of Bony Vaults in Full
Fixed and Removable Bridge Dentures, 39.
Work, 173.	Root Canal Filling. By Edmund
Hemostatic. By F. C. Deakin, 39. Noyes, 489.
Honors in Dentistry. By A. C. Rubber Dam for Fitting Crowns.
Fones in Oral Hygiene, 519.	By R. Dickson, 92.
Indirect Inlays. By H. Van Vai- Ruptured Anurysm of Tongue. By
kenberg, 441.	F. C. Sabin, 438.
Indirect Casting Inlays. By J. C. Salable and Unsalable Teeth. By
Smedley, 290.	T. H. Hinman, 393.
Schevalier of the Legion of Honor.	What is the Value of the Decidu-
By H. C. Wheeler, 134.	ous Teeth? By J. E. Gurley, 484.
Scare Advertising. Editorial in Why My Patients Come Back? 437.
Cosmos, 493.
Significance of Curable and Dead	-dwt)
Teeth. By T. P. Hinman, 395.	BIOGRAPHIC
Silk Melsh for Denture Adhesion. Baird, G. B., 384.
By C. B. Kelley, 87.	Barnes, Henry, 17.
Sizing Up the Quacks, 291.	Best and Waldron, 140-191.
Spirochetes in the Buccal and Blake, V. R., 90.
Trachea-Bronchial Regions. By Brown, R. K., 153.
H. Violla, 443.	Bogue, E. A., 96.
Sterilization of Surgical Instru- Boynton, E. F., 93.
ments. By S. S. Sanderson,	Brophy, T. M., 432.
M.D., 442.	Bryant, L. C., 428.
Sterilizing Cotton Attached to	Bunting, R. W., 302.
Broaches. By F. 0. Ormiston,	Buckley, J. P., 68.
294.	Bremner, D. K., 15.
Sterilizing Pyorrhoea Pockets. By	Carlson, W. J., 427.
K. S. Perry, 486.	Campbell, D. D„ 173.
Suggestions for Cleaning Gold	Carmichael, J. P., 218.
Nuggets. By L. R. Hess, 237.	Clapp, G. W., 54, 113, 118, 281,
Teeth and Health, 586.	354.
Teeth and Tuberculosis. Bv H. Chappelle, W. F., 360.
Brady, 242.	’	Cox, G. C., 392.
Teeth and Pregnant Women. By Davis, L. L., 431.
Harry Wexler, 91.	Dickson, R., 92.
The Accepted Filling Materials. Dittmar, G. W., 113, 430.
By G. M. Hollenback, 187.	Dresch, J. T., 292.
The Increasing Occurrence of Eby, T. B., 324.
Gangrenous Ulcerations, 588.	Endleman, J., 139.
The Choice of Anesthetics in Ex-	Elisberger, M., 366.
traction. By B. H. Harms, 239.	Epstein A. K., 418, 436.
The Technique of Pulp Capping.	Essig, N. S., 2.
By L. G. Noel, 396.	Frankel, II. G„ 272.
The Gangrenous Pulp in a De-	French, A. B., 34 .
ciduous Tooth. By J. E. Gurley,	Fones, A. C., 519.
485.	Gauley, W., 443.
The Old Ways. By Walt Mason, Gebhart, J. C., 331.
295.	'	•	Glassburg, J. A., 220.
The Sucker List. Oral Hygiene, Giffin, W. A., 103.
134.	Gurley, J. E., 484, 485.
To Polish Rubber Dentures. By Lero, A. L., 204.
F. J. Patterson, 340.	Lurie, W., 444.
Treating an Exposed Pulp. By Lowrey, E. P., 29.
Charles A. Sweet, 519.	Lyons, O. J., 260.
Treatment of Infected Dentine.	Harms, B. H., 6.
By Best and Waldron, 140.	Hartzell, T. B., 77, 265.
Unclean Mouth a Health Menace, Hess, L. R., 237.
294.	Hess, A. F., 375.
Up to Six Years, 90.	,	Hinman, T. R., 393, 395.
Value of Correct Radiographic	Hinkins, J. E., 426, 434.
Interpretations. By Dr. McCall,	Hollenback, T. M., 187.
344.	Hollister, F. W., 338.
Hopkins, A. S., 82.	ANNOUNCEMENTS
Hvatt, T. P., 26. 284, 287.	.	. n . .
Jaffer J II 324	American Dental Association, -267.
Jarvie Wm.’ 97.	American Institute of Dental
Johnson. C. N., 429.	Teachers, 47, 243, 497.
Kelley, E. B., 87.	American Society of Orthodontists,
Kemper, J. W., 103.	245-
Kent, E. N., 508.	American Academy of Periodon-
Kirk, E. C., 171.	tists, 245.
Olsen. C., 439.	American Dental	Society of
Ormiston, F. A., 294.	Europe, 243.
Ottolengui, R., 480-969.	American Society of Oral Surgery
Owen, E. B., 211.	and Exodontists, 245.
Little, C. B., 21.	Colorado State Board, 195.
Long. S. L. N., 276.	Delta Sigma Delta Fraternity, 267.
Mason, W., 295.	Dental Laboratory Association,
McCall, J. O., 127.	267.
McCoy, J. D., 524.	Dewey Orthodontia Society, 47.
Marshall, J. A., 294.	Detroit Resolution, 94.
Monson, G. S., 317.	Tndex Dental Periodicals, 497.
isobbs, A. H., 120.	ln. . o, .	’
Noel. L. G„ 396.	I11,no,S State SocietX’ 19;1-
Noves. E., 489.	Institute Orologv, 497.
Perry, K. S., 486.	Military Dental Surgeons, 245.
Pullen, H. A., 523.	Michigan Examining	Board,	496.
Rattner, M., 253.	Montana Examining	Board,	522.
Rogers, L., 103.	Massachusetts State	Society,	195.
Pm.sp Jp	Minnesota State Board, 47.
Rost. B. H.,184.	New York Rtate Board’ 95-
Roach, F. E., 433.	New York Dental College, 245.
Robertson, J. D., 487.	National Board of Examiners, 243,
Rowley, W. N., 292.	207.
Rvan, E. P. R., 163.	National Dental Faculties, 243,
Sabin, F. C., 438.	297.
Sanderson, E. S., 44.	National Dental Association, 243,
Schwimmer, A. A., 438.	267.
Smedley, V. C., 290.	National Society of Prosthetists,
Snow, G. B., 296.	243, 267.
Sixteen Years, 90.	National Library and Museum, 243.
Strang, O. M., 96.	Northern Ohio Dental Society, 243.
Sweet, C A., 509	Public HeaIth Workers, 245.
Togo^S 5 °	"	Tennessee State Society, 95.
Walker A. S. 61	National Society of Sororities, 243.
Wexler,’ H., 91.	Psi Omega Fraternity, 298.
Wheeler, H. L., 134.	Zi Psi Phy Fraternity, 267.
Wilkinson, W. W., 227.	Pacific Coast Orthodontal Society,
Van Valkenberg, H., 441.	298.
"Violle, H., 441.	Texas State Meeting, Special, 94.
BOOKS	OBITUARY
Index of Periodic Dental Litera- Clinton W. Strang, 96.
ture. By Arthur D. Block, 147. Edward A. Bogue, 96.
Orthodontic Impressions and Casts. Wm. Jarvey, 97.
By Herbert A. Pullen, D.M.D., Henjy D	243.
ta	j za i -o	i t> Robert E. Hastings, 244.
Dental and Oral Radiography. By w v Berm Ames 299
James D. McCoy, M.S., D.D.S.,	„ „Te.lgen ,“es’
524	George H. Wilson, 347.
Modern Dentistry for the Laity. Levi C. laylor, 349, 456.
By Alfred A. Crocker, 146, 498. W. E. Boardman, 455.
ANNOUNCEMENTS
Alumni Society of the Dewey School of Orthodontia.—
The next annual meeting of this ‘society will be held on
April 27-28 at the Edgewater Beach Hotel, Chicago. The
usual high standard of the meetings of this society will be
maintained. All interested in orthodontia are cordially
invited to attend these meetings.—George F. Burke, Sec.,
741-43 David Whitney Bldg., Detroit, Mich.
American Institute of Dental Tea-chers will hold its
annual meeting -January 23 to 25 at Montreal, Canada.
Minnesota State Dental Society will meet in Minneap-
olis, February 22-25, 1922.
Industrial Companies which have
completed their medical service by
the addition of a dental department
THERE have been many requests for our printed list of com-
panies using Dental Service. The book “Modern Dentistry
for the Laity” will soon appear with a corrected and up-to-date
list of all such companies.
The following is the list as it appears at the time of going
to press. Get your order in early for a copy of the new edition
of “Modern Dentistry for the Laity.”
Adamsville, Alabama—	Denver, Colorado—
Docena Iron Works.	The Colorado Fuel & Iron Co.
Tennessee Coal & Iron Co., We- Daniels & Fisher Store Co.
nonah Mines.	Gates Rubber Co.
Tennessee Coal & Iron Co, Ish- Puebl Colorado_
kooda Mmes.	The Colorado Fuel & Iron Co.
Acipo, Alabama—	Trinidad Colorado—
American Cast Iron Pipe.	The c^lorado Fuel & Iron Co.
Alabama City, Alabama	New Haven, Connecticut—
Dwight Mig. Co.	Winchester Repeating Arms Co.
Birmingham Alabama-	Milford, Delaware-
American Cast Iron Pipe Co.	The L D Caulk Co
Tennessee Coal & Iron Co.
„	,	Wilmington, Delaware—
Bessemer, Alabama—	Dupont Powder Co.
Tennessee Coal & Iron Co, Mus- Jo/eph Bancroft & Sons Co.
coda Works.	Electric Hose Co.
Chickasaw, Alabama-	New HaVen, Connecticut-
Chickasaw Shipbuilding	Co.	Kolynos Co.
Ensley, Alabama—	L. Candee Co.
Tennessee Coal & Iron Co, Bay-
view Mines	Stamford, Connecticut—
Westfield CRnic.	Yale & Towne W Co-
Edgewater, Alabama-	Sozu,th Manchester, Connecticut-
Edgewater Works.	Cheney Bros. Co.
Fairfield, Alabama—	Atlanta, Georgia-
Tennessee Coal & Iron Co.	Fulton Bag & Cotton Mills.
Johns, Alabama—	Chicago, Illinois—
Tennessee Coal & Iron Co.	Morris & Co.
Davenport, California—	Montgomery & Ward	Co.
Santa Cruz Portland Cement Co.	Yrmo^r &
.	The Crane Co.
Fresno, California—	The Biamond Match	Co.
California Associated Raisin Grow- Hart> Schaffner & Marx.
ers‘	Kabo Corset Co.
San Francisco, California—	Sears, Roebuck & Co.
The Emporium.	The International Harvester Co.
Peoria, Illinois—	Kansas City, Missouri—
The Avery Co.	Jones Store Co.
Montgomery & Ward.
Indiana Harbor, Indiana—	National Lamp Works.
The Inland Steel Co.	_
St. Louis, Missouri —
New Orleans, Louisiana—	National Lamp Works.
D. H. Holmes Co.	Union Electric Light & Power Co.
Maison Blanche, Inc.	Monsanto Chemical Works.
Stix, Baer & Fuller Dry Goods Co.
South Brewer, Maine—	Century Electric Co.
The Eastern Mfg. Co.	B. Nugent & Bros. Dry Goods Co.
Boston, Massachusetts—	Concord, New Hampshire—
The Hood Rubber Co.	Union District Dental Clinic.
Filene Co-operative Association.
Manchester, New Hampshire—
Watertown, Massachusetts—	The Amoskeag Co.
Hood Rubber Co. (Factory).	,T ,
Nashua, New Hampshire—
Framingham, Massachusetts—	Nashua, City Dental Clinic.
The Dennison Mfg. Co.	Maginnis Cotton Mills.
Chicopee Falls, Massachusetts—	Harrison, New Jersey—
Fisk Rubber Co.	Harrison Lamp Works.
A. G. Spalding Co.	The General Electric Co.
Pittsfield, Massachusetts—	Hurley, New Mexico—
General El. Co.	Chino Copper Co.
Springfield, Massachusetts—	Brooklyn, New York—
Am. Bosch Magneto Co., Bright- Schrader & Sons.
wood Station.	, _T	,
National Equipment Co.	UJ ukin^6"
Holyoke, Massachusetts—	American Radiator Co.
Farr Alpaca Co.	Buffalo Miniature Lamp Works.
Pierce-Arrow Motor Car Co.
Lynn, Massachusetts—
General Electric Co.	Johnson City, New York—
Endicott-Johnson Co.
Worcester, Massachusetts—	E.-J. Workers Medical & Relief.
The Norton Co.	~	„ tr ,
McGregor, New York—
Wekter, Massachusetts—	Metropolitan Life Insurance Co.
S. Slater & Sons Co.	New York City, New York—
Wellesly Hills, Massachusetts-	’“rS'01“1 GarnlCnt Workl!rs’
Babson Statistical Organization.	Lord & Taylor
Detroit, Michigan—	Macv Mutual Aid Assn.
The Ford Motor Car Co.	Î?1?68 ,uCreery & Co.
Solvay Process Co.	Metropolitan Life Insurance Co.
New York Telephone Co.
Kalamazoo, Michigan—	John Wanamaker Store.
Kalamazoo Stove Co.	Colgate & Co.
Sanatorium.
Minneapolis, Minnesota—	Niagara Falls New York—
The Washburn-Crosby Co.	nm v	fri +	™	n
The Northwestern Knitting Co.	Oldsbury Electro Chem. Co.
National Lamp Co.	Rochester, New York—
Munsingwear Corporation.	The Bousch & Lomb Co.
Schenectady, New York—	Pittsburgh, Pennsylvania—
General Electric Co.	The Armstrong Cork Co.
..	.	The H. J. Heinz Co.
™on> ?hl0~	„ -o ,,	„	Lee S. Smith & Son Co.
The Firestone Tire & Rubber Co.	Kauffman's “The Big Store.”
the B. F. Goodrich Co.	Kaufmann’s, The Big Store.
Cincinnati, Ohio—	™	™
The Cincinnati Milling Machine Co. Reading, Pennsylvania—
The R. K. LeBlond Machine Tool Co. Berkshire Knitting Mills.
The Worthington Pump Co.	Berkshire Knitting Machine Co.
The Lunkenheimer Co.	Bristol, Rhode Island_
Cleveland, Ohio—	National India Rubber Co.
The Bailey Co.	z. x ,	™ T ,
The Joseph & Feiss Co.	Central Falls, Rhode Island—
The Kaynes Co.	Rhode Glass Division (Natl. Lamp
National Lamp Works of General	Works, General Electric Co.).
EL Co (Parent Co. of all Natl. Esmond Rhode Isiand_
Lamp Works.)	Esmond Mills.
H. & E. Blouse Co.
Columbus, Ohio-	Bosevaine, Virginia-
Jeffries Mfg. Co.	Pocahontas Juel Co.
Dayton, Ohio—	Itman, West Virginia—
Dayton Electric Laboratories Co.	Pocahontas Fuel Co.
The Dayton Metal Products Co.	_ , . .
The National Cash Register Co.	Jenkinjones, West Virginia
Domestic Engineering Co.	1 ocahontas Juel Co.
Hamilton, Ohio—	Maybeury, West Virginia—
The Hooven-Owens-Rentschler Co.	Pocahontas Fuel Co.
Newcomerstown, Ohio—	War, West Virginia—
J. B. Clow Co.	Williams Pocoliontas Coal Co.
Warren, Ohio	Appleton, Wisconsin—
National Lamp Works of General Kimberlv-Clark Co
El. Co.
.	Kohler, Wisconsin—
Youngstown Ohio-	Kohler Mfg. Co.
National Lamp Works of General
El. Co.	Milwaukee, Wisconsin—
Erie, Pennsylvania—	Phoenix Knitting Mills.
Erie Foro-e Co.	Cutler-Hammer Mfg. Co.
j i v -n i •	Chain Belt Co.
Philadelphia Pennsylvania-	Wisconsin Bridge Works.
Powers, Weightman, Rosengarten
Co.	Neenah, Wisconsin—
John Wanamaker.	Kimberlv-Clark Co.
The S S. White Dental Mfg. Co. Ni	wisconsin_
Lit Brothers.	» . ’ . n i
J. G. Brill Co.	Kimberly-Clark Co.
John B. Stetson Co.	Toronto, Canada—
The American Pulley Co.	T. Eaton Co.. Ltd.
				

